# The Hypodermic Use of Cardiac Stimulants

**Published:** 1904-03-12

**Authors:** 


### THE HYPODERMIC USE OF CARDIAC STIMULANTS.

The important therapeutic properties of bodies of the digitalis group is well recognised. Their use when given by the mouth has been established by wide clinical experience, but the employment of hypodermic means of administration has lead to some discordant results.

The scientific basis of the hypodermic administration of these bodies has been established by Fraenkel. In a recent article (*Arch. f. Exp. Path und Pharm. B.* 51, 1) he gives the results of a long series of experiments. He finds that there was no evidence of any immunity conferred by a long series of doses, but on the contrary the cumulative action of these drugs was quite evident. The time at which the cumulative action causes intoxication depends directly upon the magnitude of the doses and the shortness of the interval between the doses. In these experiments, with hypodermic administration of the drug, the individuality of the animals plays a very small part.

For each one of the bodies investigated—digitalin, digitoxin, and strophanthin—a certain daily dose can be ascertained, the introduction of which causes definite therapeutic effects. If this amount be exceeded, a toxic accumulation results with all of these substances. But the tendency towards toxic accumulation differs with the separate bodies. For example, 0·08 milligramme of digitoxin and 0·48 milligramme of digitalin are physiologically equivalent, and can be reckoned as the single lethal doses of the two bodies. There is a marked difference, however, in the case of repeated doses. Digitoxin, in doses only one-third of this single dose, poisons after the third daily injection, and makes the animal very ill for nine days. On the other hand, one-half of the single lethal dose of digitalin can be given daily for a week without bringing on any symptoms of poisoning.

Also it is found that the daily interval between the digitoxin injections must be greater than for digitalin, if one would avoid intoxication—*i.e.* digitoxin accumulates more readily. Again, with the single dose of digitoxin the interval between the active and the deadly dose is so small that pulse-slowing produced by a single injection was not obtained without at the same time causing a fatal intoxication. In contrast to this, digitalin and strophanthin both produced the effect on the pulse quite successfully without a fatal result.

All "digitalis bodies" appear to possess the property of cumulative activity. It occurs as easily with the water-soluble strophanthin and also with helleborein. With the less soluble substances there is a delayed onset of their action, but the accumulative property stands out more strongly. That the cumulative action is not in direct relation to the solubility of the drug is shown by the strong after-effects and cumulative action of the soluble strophanthin.

One must, therefore, draw the conclusion that this particular property depends not so much on the rate of excretion from the blood as upon the firmness of combination with the susceptible tissues of the heart. Stokiss and Heide arrived at a similar result from their experiments with the very soluble helleboreins. Also the rapidity with which the heart takes up the active principle in the case of these bodies depends on the firmness of the combination; and this also determines, in some degree, the time that the effect lasts.

The alterations of the heart's activity, the increased output, and the slowing of the rate, usually last for some time. The onset of the effect, on the other hand, differs widely with the various preparations. With strophanthin the circulation, after a few hours, begins to show the effect of a therapeutic dose.

A dose of digitalin, of equivalent physiological value, does not produce its effect for 24 hours; and with digitoxin until an even later period, up to 60 hours. These facts have a practical and clinical importance. If one aims at rapid action, strophanthin is the most suitable. Also in the case of strophanthin a dangerous overdose is more easily avoided—a small transgression of the therapeutic limit is made evident in a few hours by the phenomena of commencing intoxication. For this reason strophanthin and digitalin appear to be more suitable for administration as cardiac tonics than digitoxin.

For a short energetic effect, digitoxin administered in small doses appears suitable, but not for continued administration. For an immediate effect on the circulation, strophanthin, from the greatest rapidity of its action, is to be preferred. Yet even with this drug the fear of accumulation must always be borne in mind. The clinical fact of greater importance is the delay that takes place in the action of these drugs when administered hypodermically, and this is particularly marked with digitoxin, though of considerable duration with digitalin. These experimental results should be carefully considered when the necessity for their administration arises.

